# Distorted Sex Ratios: A Window into RNAi-Mediated Silencing

**DOI:** 10.1371/journal.pbio.0050303

**Published:** 2007-11-06

**Authors:** Patrick M Ferree, Daniel A Barbash

## Abstract

Some species of*Drosophila* have unequal ratios of males to females, and now two genes--one responsible for such sex-ratio distortion and one that suppresses it--have been identified in one of these species.

In 1925, Gershenson started laboratory cultures from 19 female Drosophila obscura that were collected from a forest near Moscow. After recounting his difficulties raising the flies (partial success achieved with a diet of potatoes and fermented raisins), he noted that progeny from most cultures contained an approximately equal sex ratio [1]. Several cultures, however, yielded progeny with highly skewed ratios, such as one group with 87 females and only 7 males. These “deviations from the normal sex-ratio were so considerable that it seemed impossible to explain them by accidental causes,” he wrote. Similar observations had been made by others, but Gershenson went on to perform a number of experiments and reached three important conclusions. First, sex-ratio distortion (referred to hereafter as *sex-ratio*) was associated with the X chromosome. Second, the expression of the phenotype was sex-limited, because it only occurred in the progeny of males carrying the causal X chromosome. And third, the low numbers of males did not appear to be caused by preferential death of male zygotes or their transformation into females. Rather, he concluded that “the greater part of the spermatozoa determining the development of males do not participate in fertilization”. Because females have two X chromosomes and males are XY, he further suggested that either Y-bearing sperm are less frequently produced by affected males than X-bearing sperm, or that Y-bearing sperm are less capable of achieving fertilization.

Numerous additional examples of *sex-ratio* have since been reported in other Drosophila species, but the identity of the causal genes has remained elusive. In this issue of *PLoS Biology*, Yun Tao and colleagues report the discovery and identity of an X-linked *sex-ratio* distorter from Drosophila simulans called *Dox* (*Distorter on the X*) [2]. In a second paper [3] they describe the identification of a dominant suppressor of *Dox* called *Nmy* (*Not much yang*).

The close association of distorting and suppressing genes, though not appreciated by Gershenson, is key to understanding the genetic basis and evolutionary dynamics of *sex-ratio* systems. The long-term prospects of sexually reproducing populations that contain predominantly one sex are dire, and theory (commonly attributed to R.A. Fisher, but see [4] for an alternative attribution) suggests that an equal sex ratio is generally the most stable ratio over evolutionary time. Genes causing *sex-ratio* are therefore selfish genes, good at promoting an increase in their own frequency while potentially driving their host species to ruin. The host species is thus predicted to evolve suppressing alleles in order to maintain an equal sex ratio. This battle between *sex-ratio* distorters and suppressors creates a genetic conflict and may lead to continual cycles of distortion and suppression.

One then expects that *sex-ratio* phenotypes may arise in progeny of crosses between populations or species, as distortion genes segregate away from their corresponding suppressors. Such phenotypes occurred when segments of the Drosophila sechellia genome were introgressed into its sister species, D. simulans [5]. Skewed sex ratios were observed in ~10% of such introgression lines. Tao et al. [3] chose one of these lines for further characterization, reasoning that an introgressed D. sechellia region was displacing a D. simulans *sex-ratio* suppressor and thus unleashing a D. simulans distortion gene. They found the suppressor gene, *Nmy*, but discovered that the absence of the suppressor was caused by a mutant allele, *nmy*, that is segregating within D. simulans populations, rather than being due to introgression from D. sechellia. While analyzing *nmy*, they also found a D. simulans strain that did not produce an altered sex ratio even when homozygous for *nmy* [2]. This strain turned out to carry a mutant allele, *dox*, that is incapable of distortion. The discovery of these mutant alleles *dox* and *nmy* allowed Tao and colleagues to map the corresponding genes to single-gene resolution.

*Dox* appears to be a transposition from a parental gene named by the authors as *MDox* (*Mother of Dox*). *MDox* partially overlaps the 3′ end of another gene, *CG32702*, but is transcribed on the other strand. Both *MDox* and *Dox* have limited protein-coding potential, and the longer hypothetical open reading frames do not match any known protein sequences. Determining whether the distorting activity of *Dox* is mediated via an RNA or protein product will require future experimental analysis. Most strikingly, Tao et al. [3] found that the suppressor *Nmy* appears to have originated as a retrotransposed duplication of *Dox*. A further duplication generated an inverted repeat in *Nmy*, and deletion of one of these repeats creates a nonsuppressing *nmy* mutant allele. From these observations, Tao et al. propose that small interfering RNAs (siRNAs) generated from the *Nmy* hairpin sequence silence *Dox* via the RNA interference (RNAi) pathway (Figure 1).

**Figure 1:**
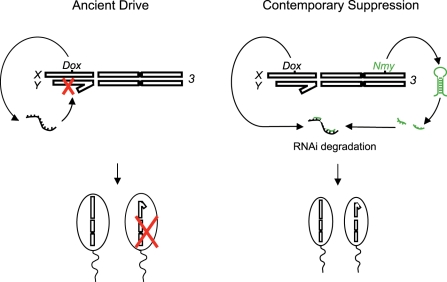
Mechanistic and Evolutionary Model for *sex-ratio* Distortion The X-linked *Dox* gene first evolved to target an unknown component of the Y chromosome, so that Y-bearing sperm fail to develop. This leads to an increased transmission frequency of the *Dox*-bearing X chromosome and a female-biased sex ratio. It remains unclear whether *Dox* is an RNA or protein-coding gene. Later, a transposition of *Dox* to Chromosome 3 created the *Nmy* gene. siRNAs produced from the double-stranded hairpin of *Nmy* target the homologous region of *Dox* for degradation via the RNAi pathway. As a result, Y-bearing sperm develop normally, and X-chromosome meiotic drive is suppressed. The model depicts a pre-meiotic germ cell, but the cellular manifestation of distortion occurs during nuclear condensation and maturation of sperm. Only the sex and third chromosomes are shown.

These exciting studies beg for a deeper understanding of both the mechanistic basis and evolutionary consequences of *sex-ratio*. The foremost mechanistic question is how *Dox* incapacitates Y-bearing sperm. It will then be of great interest to compare the mechanism of *Dox*-mediated *sex-ratio* with other distortion systems. Tao et al. argue that other systems previously described in D. simulans are genetically independent. One of these has been intensively studied by Montchamp-Moreau and colleagues, and the distortion genes have been mapped to high resolution (reviewed in [6]). Hints of additional distortion systems and extensive polymorphism within D. simulans that modify *sex-ratio* are scattered throughout the articles of Tao et al. These findings therefore suggest that cycles of *sex-ratio* followed by suppression may be quite frequent, and thus may have a marked impact on genome evolution. If so then these reports will certainly reignite long-standing and contentious arguments about whether and how *sex-ratio* genes contribute to hybrid male sterility and speciation [7–13]. Much of this debate has centered around the question of whether *sex-ratio* genes are a predominant cause of Haldane's rule, the observation that the heterogametic sex (XY males and ZW females) more often suffers from hybrid incompatibilities than the homogametic sex (XX females and ZZ males).

These studies may also provide insight into the molecular basis of autosomal distortion systems. Tao et al. [3] suggest that a failure in RNAi-mediated silencing may explain the mechanism of a well-known autosomal distortion system, *Segregation Distorter* (*SD*). Here distortion is caused by *Sd*, a partial duplication of RanGAP, a GTPase that is found primarily in the cytoplasm and is required for nuclear transport. *Sd* encodes a truncated RanGAP that is enzymatically active but mislocalizes to the nucleus due to the deletion of a nuclear export signal [14,15].

Unlike in the *sex-ratio* system, the locus targeted by *Sd* is known. This locus, called *Responder* (*Rsp*), consists of an array of 240-bp satellite repeats. Sensitivity to the distorting effect of *Sd* is proportional to copy number of the satellite repeat: *Responder*-insensitive (*Rsp* ^i^) alleles have as few as 20–30 copies and are not distorted by *Sd*, whereas *Responder*-sensitive (*Rsp* ^S^) alleles carry up to 3,000 copies [16]. Because the *Sd* and *Rsp* loci are closely linked on the second chromosome, *Sd* alleles only persist in populations if they remain linked in *cis* to an *Rsp* ^i^ allele (Figure 2). Distortion is induced by *Sd* against a Chromosome 2 homolog that carries *Rsp* ^S^. In such males, the nuclei of half of the gametes—those of genotype *Sd* ^+^, *Rsp* ^S^—fail to undergo normal chromatin condensation, which blocks their maturation into individual spermatocytes [17]. Chromatin condensation phenotypes were observed by Tao et al. [3] in Y-bearing sperm from *Dox; nmy* males, suggesting that a similar developmental process may be disrupted to cause distortion in this *sex-ratio* system.

**Figure 2:**
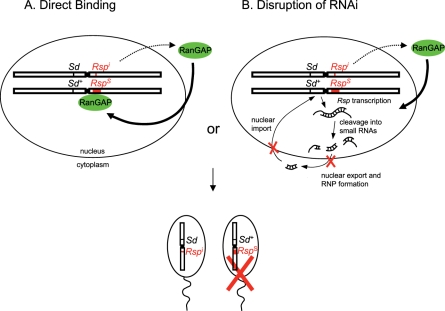
Two Models for the Role of *Responder* (*Rsp*) in *Segregation Distortion* (*SD*) The *Sd* gene encodes a truncated RanGAP, which mislocalizes to the nucleus. (A) In a previous model [14], nuclear RanGAP binds abnormally to *Rsp* satellite repeats on Chromosome 2 during spermatogenesis. Chromatin condensation is disrupted in chromosomes carrying a high number of repeats (*Rsp* ^S^), resulting in developmental failure of sperm bearing these chromosomes [16]. (B) Alternatively, *SD* is caused by disruption of an RNAi-dependent silencing process as suggested by Tao et al. [3]. Mislocalized RanGAP disrupts proper nuclear transport of small *Rsp*-derived RNAs and ribonucleoprotein (RNP) complexes that are required to repress the *Rsp* satellites. As a result, proper heterochromatic repression of *Rsp* ^S^ is disrupted, causing defects in chromatin condensation and loss of *Rsp* ^S^-bearing sperm.

One of the longstanding mysteries of *SD* is the link between mislocalized RanGAP and *Responder*. A previous model has proposed that RanGAP directly binds to *Responder* satellite DNA to disrupt chromatin condensation [14] (Figure 2). However, Tao et al. [3] propose an intriguing alternative hypothesis: that *SD*, like *sex-ratio*, occurs through an RNAi mechanism (Figure 2). Recent studies have shown that noncoding, highly repetitive DNA sequences called satellites as well as repetitive mobile genetic elements are silenced by the repeat-associated small interfering RNA (rasiRNA; also called Piwi-associated RNA or piRNA) pathway in a number of organisms including Drosophila [18–22]. Transcripts made from these typically heterochromatic sequences are processed into 22–30-bp double-stranded RNAs by proteins including Piwi and Aubergine, and are transported into the cytoplasm where they are complexed with other proteins such as Argonaute-3. Current models suggest that these ribonucleoprotein complexes are then shuttled back into the nucleus where they recruit histone methyltransferases like SU(VAR)3-9 to the sequences homologous to those from which they were derived. This sequence-dependent association induces methylation of lysine 9 on histone-3, recruitment of the heterochromatin protein-1 (HP1), and transcriptional silencing of these regions.

Tao et al. [3] suggest that the mislocalized RanGAP encoded by *Sd* impairs transport of *Responder* RNAs into the nucleus, causing a failure of silencing of the *Responder* locus and subsequent loss of proper chromatin condensation. High copy number of the *Responder* satellite would cause *Rsp* ^S^ alleles, because they create a larger area of decondensed chromatin. According to this hypothesis, condensation defects in *SD* nuclei are expected to: (1) initiate on, and be confined to, the second chromosome and (2) be modified by mutations in the rasiRNA pathway. Both of these predictions are experimentally testable.

We suggest that a similar failure in RNAi-mediated silencing of pericentromeric satellite DNA, which is adjacent to the centromere, may cause hybrid lethality in Drosophila. Hybrid daughters of D. simulans females crossed to D. melanogaster males die as embryos. Sawamura et al. [23] discovered that lethality depends on the D. melanogaster X-chromosomal locus *Zygotic hybrid rescue* (*Zhr*). Sawamura et al. [24] proposed that *Zhr* may correspond to a 359-bp repeat belonging to the 1.688-g/cm^3^ family of satellites, which is located on the D. melanogaster X chromosome. After examining multiple *Zhr* ^+^ and *Zhr* ^−^ chromosomes, they observed that the amount of satellite did not perfectly correlate with hybrid lethality and concluded that *Zhr* does not correspond to the 359-bp satellite. In general, however, lethal genotypes did contain high amounts of the satellite, suggesting the possibility that a threshold amount of satellites is needed to induce lethality. The 359-bp satellite was recently shown to be silenced in the ovary by the RNAi machinery [25]. We propose that maternally contributed small RNAs deriving from the 359-bp repeat are required to silence paternally inherited copies of the repeat in the developing embryo. These maternal RNAs will be particularly critical during early embryonic cell cycles, before zygotic transcription begins. D. simulans does not appear to contain the 359-bp repeat found on the D. melanogaster X chromosome [26]. Therefore, the 359-bp satellite on the paternally inherited D. melanogaster X chromosome will be de-repressed in early hybrid female embryos (Figure 3). This hypothesis explains why lethality is female-specific, because hybrid sons carry only the D. simulans X chromosome.

**Figure 3:**
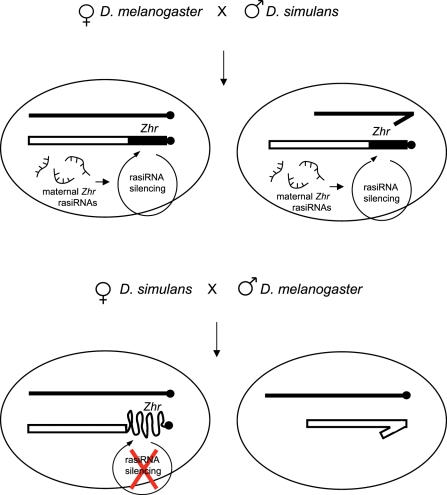
Model for the Role of RNAi in Hybrid Embryonic Lethality Hybrid embryos produced from D. melanogaster mothers and D. simulans fathers inherit *Zhr*-derived rasiRNAs from the maternal cytoplasm. These small RNAs are required during the early embryonic divisions for proper silencing of the *Zhr* locus. In contrast, hybrid embryos from D. simulans mothers and D. melanogaster fathers do not contain maternally loaded *Zhr* rasiRNAs. As a result, *Zhr* is not silenced in hybrid females carrying the D. melanogaster X chromosome, resulting in heterochromatin decondensation and embryonic death. Hybrid male embryos from this cross do not carry the D. melanogaster X and thus are viable. We suggest that the 359-bp, 1.688-g/cm^3^ satellite corresponds to *Zhr* (see text for details).

Other factors may contribute to this embryonic hybrid lethality, and our hypothesis raises further questions of whether other repeat classes that are specific to either D. melanogaster or D. simulans are de-repressed in their hybrids. Similar RNAi-based mechanisms have also been suggested to maintain silencing of transposable elements (TEs). Hybrid dysgenesis occurs when females lacking a TE family mate with males from a different strain containing the TE. The TEs become active in the hybrid progeny, and their mobilization can lead to increased mutation rates, chromosomal rearrangements, and sterility (for an early review see [27]). A striking example of the maternal specificity comes from a study of Arabidopsis, where molecular polymorphisms distinguishing different families of the ATHILA retrotransposon were used to demonstrate that only paternally inherited copies are de-repressed in interspecific hybrids [28]. A link to rasiRNA production was suggested in a D. virilis hybrid dysgenesis system (marked by abnormal gonadal development), where maternal expression of small RNAs derived from the Penelope retrotransposon correlates with suppression of dysgenesis [29]. Extensive sequencing of rasiRNAs in D. melanogaster has recently led to the suggestion that RNAi-mediated silencing may regulate many different TE families [22]. The aforementioned Arabidopsis study reported additional defects in imprinting in hybrids, and the authors suggest that misregulation of chromatin states may be a general cause of hybrid lethality [28]. Mechanistic details of how RNAi contributes to gene silencing and heterochromatin establishment and maintenance are currently the topic of much exciting research (reviewed in [30,31]). This first mechanistic glimpse of a *sex-ratio* distortion system provided by Tao and colleagues suggests that RNAi-mediated regulation, and its failure, may have important implications for understanding fundamental problems in evolutionary genetics and speciation.

## Abbreviations

rasiRNA: repeat-associated small interfering RNA
RNAi: RNA interference
*SD*: *Segregation Distorter*
TE: transposable element
